# Genome-Wide Association Study of Major Agronomic Traits in Foxtail Millet (*Setaria italica* L.) Using ddRAD Sequencing

**DOI:** 10.1038/s41598-019-41602-6

**Published:** 2019-03-22

**Authors:** Vandana Jaiswal, Sarika Gupta, Vijay Gahlaut, Mehanathan Muthamilarasan, Tirthankar Bandyopadhyay, Nirala Ramchiary, Manoj Prasad

**Affiliations:** 1 School of Life Science, Jawaharlal Nehru University, New Delhi, 110067 India; 2 Department of Plant Molecular Biology, University of Delhi South Campus, New Delhi, 110021 India; 3 National Institute of Plant Genome Research, New Delhi, 110067 India; 4 ICAR-National Research Centre on Plant Biotechnology, LBS Centre, Pusa Campus, New Delhi, 110012 India

## Abstract

Foxtail millet (*Setaria italica*), the second largest cultivated millet crop after pearl millet, is utilized for food and forage globally. Further, it is also considered as a model crop for studying agronomic, nutritional and biofuel traits. In the present study, a genome-wide association study (GWAS) was performed for ten important agronomic traits in 142 foxtail millet core eco-geographically diverse genotypes using 10 K SNPs developed through GBS-ddRAD approach. Number of SNPs on individual chromosome ranged from 844 (chromosome 5) to 2153 (chromosome 8) with an average SNP frequency of 25.9 per Mb. The pairwise linkage disequilibrium (LD) estimated using the squared-allele frequency correlations was found to decay rapidly with the genetic distance of 177 Kb. However, for individual chromosome, LD decay distance ranged from 76 Kb (chromosome 6) to 357 Kb (chromosome 4). GWAS identified 81 MTAs (marker-trait associations) for ten traits across the genome. High confidence MTAs for three important agronomic traits including FLW (flag leaf width), GY (grain yield) and TGW (thousand-grain weight) were identified. Significant pyramiding effect of identified MTAs further supplemented its importance in breeding programs. Desirable alleles and superior genotypes identified in the present study may prove valuable for foxtail millet improvement through marker-assisted selection.

## Introduction

Foxtail millet (*Setaria italica*) is a C_4_ self-pollinated cereal crop known to be cultivated since 5000–6000 BC on the banks of Yellow River in China^1^. The crop has major agronomic advantages in terms of being relatively cheap to cultivate, tolerant to biotic and abiotic stresses^1–4^, efficient in water use^5^ and nutritionally rich^2,3,6,7^. It is a major crop in the arid and semi-arid regions of Asia, sub-Saharan Africa and China^8^ and has increasingly emerged as one of the promising climate-resilient crops in the present decade^9^. Moreover, foxtail millet has a relatively smaller diploid genome of 510 Mb^10,11^ and is considered as an ideal C_4_ model system for genetic studies involving C_4_ photosynthesis, agronomically important stress responses, bioenergy potential^2^ among many others. Despite possessing such attractive agronomic traits, endeavours to understand, dissect and utilize the genetic diversity and generate mapping resources of the crop has been limited^12–14^. Thus, a better understanding of the genetic basis influencing the variation in agronomic traits stands to significantly augment crop improvement strategies through conventional breeding or biotechnological approaches.

In foxtail millet, linkage-based QTL mapping has been conducted for several agronomic traits including yield, grain weight, flowering days, seed number, etc.^15–17^. Linkage-based mapping suffers from poor mapping resolution, less allele mining due to the utilization of biparental population. It is also very tedious to develop mapping population. Alternatively, linkage disequilibrium (LD) based genome-wide association study (GWAS) has higher mapping resolution due to the utilization of historical recombination events available in a natural population^18^. GWAS has been widely conducted in each of the important crops and model plant systems (like wheat, rice, maize, Arabidopsis) for several traits including agronomic, quality, disease resistance, etc.^19–24^. However, in foxtail millet, only a few studies are available on GWAS^25,26^.

Advancements in technologies like next-generation sequencing (NGS), genotyping-by-sequencing (GBS) and SNP chips further improve the utility of GWAS through the development of high-density genotyping data. Further, to deal with false positives due to population structure and multiple testing in GWAS, statistical tools are being continually developed^27^. Given this, high-quality SNPs distributed throughout the foxtail millet genome were mined using Double Digest Restriction Associated DNA (ddRAD) sequencing of 142genotypes. GWAS was performed for ten agronomic traits using 10367 SNPs through FarmCPU approach. Superior alleles and genotypes identified in the present study stand to significantly facilitate the improvement of foxtail millet as a viable and efficient climate resilient crop through marker-assisted selection and other useful crop improvement programmes in the future.

## Results

### Trait distribution and correlation

Descriptive statistics including minimum, maximum, mean and standard deviation revealed a wide range of variability for each of the ten traits across 142 genotypes and summarized in Supplementary Table 1. In summary, a wide range of variability was observed; for example, day of flowering (DOF) ranged from 36–65 with mean 51.4 and a standard deviation of 5.6. Similar variability was observed in other traits including plant height (PH; mean ± SD; 138.4 ± 19.6), tiller number (TN; mean ± SD; 4.3 ± 1.2), flag leaf length (FLL; mean ± SD; 31.4 ± 6.0), flag leaf width (FLW; mean ± SD; 1.8 ± 0.4), peduncle length (PedL; mean ± SD; 19.0 ± 4.4); panicle length (PanL; mean ± SD; 14.6 ± 3.7), tiller maturity (TM; mean ± SD; 87.8 ± 8.0), grain yield (GY; mean ± SD; 12.8 ± 7.8) and thousand grain weight (TGW; mean ± SD; 2.9 ± 0.6). Frequency distribution of each trait in the population was revealed through histograms (Supplementary Fig. 1). Pearson’s correlation analysis identified that out of 45 trait-pairs (using ten traits), 22 pairs were significantly correlated (Supplementary Table 2). Out of 22 correlations, 14 were positive, and eight were negative. A maximum positive correlation (r^2^ = 0.47) was observed for FLL/FLW. However, TGW and GY were negatively correlated to the maximum extent (r^2^ = −0.716).

### Distribution of SNPs on foxtail millet chromosomes

**Figure 1:**
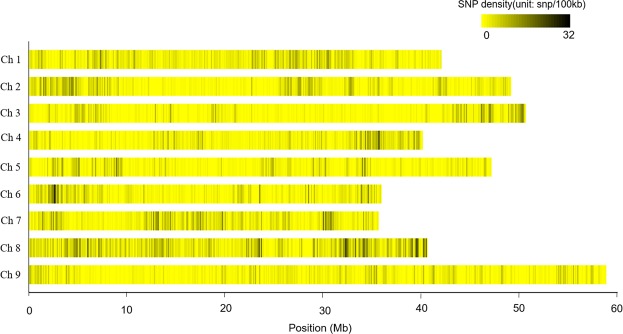
Single nucleotide polymorphism (SNP) density on nine foxtail millet chromosomes. The x-axis shows the interval distance in Mb. Window size to calculate SNP density 100 Kb.

GBS enabled the identification of ~30,000 SNPs. After filtration (removing markers with missing data <30% and minor allele frequency >5%), 12460 SNPs were selected for physical mapping on foxtail millet chromosomes. Out of 12460 SNPs, 10367 were mapped on nine major scaffolds of foxtail millet. These major scaffolds (1–9) were considered as nine chromosomes (1–9), respectively, hereafter. The mapped 10367 SNPs covered a total of 399.9 Mb of foxtail millet genome. Thus, the average SNP frequency in foxtail millet genome was observed as 25.9 SNPs/Mb. On individual chromosome, the number of SNPs ranged from 844 (chromosome 5) to 2153 (chromosome 8) (Table 1). Distribution of SNPs across the nine chromosomes is given in Fig. 1. Length of individual chromosome varied from 35.6 Mb (chromosome 7) to 58.9 Mb (chromosome 9). Maximum SNP density was observed on chromosome 8 (53.0 SNPs/Mb) and minimum on chromosome 9 (16.7 SNPs/Mb).

**Table 1:** Distribution of 10367 SNPs on nine foxtail millet chromosomes. A summary of SNP pairs showing significant linkage disequilibrium (LD) and LD decay distance on each of the nine chromosomes is also shown.

Chromosome	Number of SNPs	Chromosome length (Mb)	SNP density (per Mb)	SNP pair in LD (*p* ≤ 0.05)	Average LD (r^2^)	LD decay distance (Kb)
1	1236	42.0	29.4	21289	0.17	306
2	1216	49.1	24.8	16805	0.12	155
3	872	50.6	17.2	8466	0.09	121
4	970	40.1	24.2	14388	0.19	357
5	844	47.1	17.9	9524	0.14	350
6	923	35.9	25.7	7461	0.09	76
7	1171	35.6	32.9	13627	0.11	136
8	2153	40.6	53.0	28790	0.14	137
9	982	58.9	16.7	8136	0.10	204

### Linkage Disequilibrium

**Figure 2:**
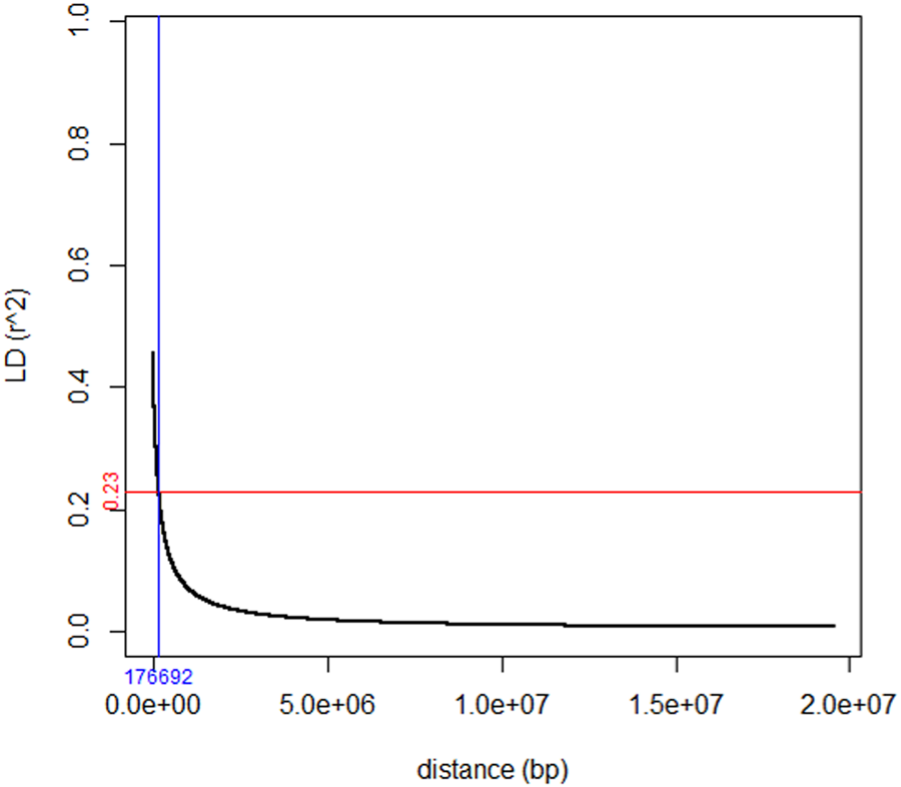
Genome-wide linkage disequilibrium (LD) decay plots. x-axis represents distance (base) between SNPs and y-axis represents LD value (r^2^; 0.0, 0.2, 0.4, 0.6, 0.8, 1.0). Horizontal and vertical lines represent half LD and LD decay distance respectively.

LD and LD decay distance in all the nine foxtail millet chromosomes is summarized in Table 1. A maximum of 28790 SNP pairs on chromosome 8 showed significant (*p* < 0.05) LD; however, a minimum of 7461 SNP pairs crossed the significance level of LD on chromosome 6. The whole genome average maximum r^2^ value found as 0.46 which dropped to its half (0.23) as distance 177 Kb; thus, considered as whole genome LD decay distance and above which LD decayed (Fig. 2). However, for individual chromosome, maximum LD decay distance was observed for chromosome 4 (357 Kb), followed by chromosome 5 (350 Kb), while chromosome 6 (76 Kb; Table 1, Supplementary Fig. 2) showed the minimum LD decay distance.

### Genome-wide marker-trait associations

**Figure 3:**
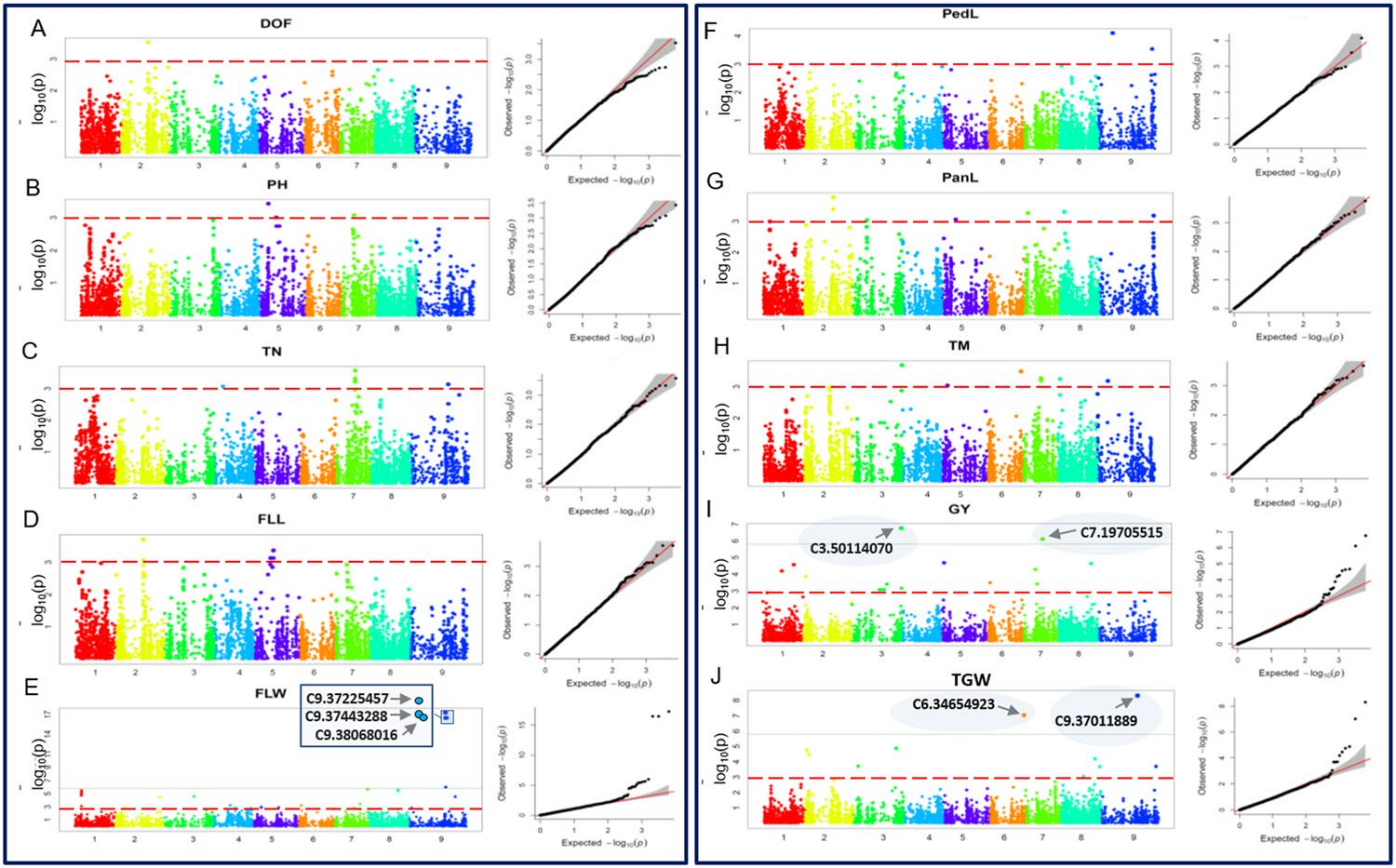
Manhattan plots and quantile-quantile (Q-Q) plots (A and J) of the GWAS results for following 10 traits including Days to flowering (DOF), plant height (PH), tiller number (TN), flag leaf length (FLL), flag leaf width (FLW), peduncle length (PedL), panicle length (PanL), tiller maturity (TM), grain yield (GY), and 1000 grain weight (TGW), respectively. Significant MTA threshold [−log 10 (p) < 10^−03^] and Bonferroni threshold are represented by dash (red) and continuous (grey) lines, respectively. x-axis represents chromosomes.

Altogether, 81 marker-trait associations (involving 79 SNPs) were identified for ten traits using FarmCPU (Table 2) with *p*-value < 0.001. Two SNPs (C9.37523364 and C7.19705515) were associated with two traits (FLW/TN and GY/TN) each, respectively. Above mentioned 81 MTAs were present on all the nine chromosomes (Fig. 3). Q-Q plots between observed and expected *p*-values of association revealed appropriate model fitting involving population structure and kinship (Fig. 3), although the power of test statistics was lower in some cases. For DOF, only one SNP (C2.27561819 on chromosome 2) was found to be associated. A maximum of 21 MTAs was identified for FLW involving all the chromosomes except chromosome 5. Most significant MTA for FLW was present on chromosome 9. For GY, 17 MTAs were identified on seven chromosomes (1–3 and 5–8), and most significant MTA was present on chromosome 3. Similarly, for TGW, 10 MTAs were identified on five chromosomes (2, 3, 6, 8 and 9). Seven MTAs identified each for three traits including FLL (chromosomes 2 and 5), PedL (chromosomes 2, 3, 5, 6, 7 and 9), and TM (chromosomes 3, 5–9). Total six, three and two MTAs were identified for TN, PH and PedL, respectively. Summary of above mentioned 81 MTAs along with chromosomal position, *p*-value, minor allele frequency and SNP effect is given in Table 2.

**Table 2:** List of significant MTAs along with contrasting alleles, chromosome, position, minor allele frequency (MAF) and SNP effect. 0.001 is considered as *P-*value cut off for significant MTAs. Earlier studies where QTLs are reported for same trait on same chromosomes are mentioned in last column.

Trait	SNP	Alleles	Chromosome	Position	−log(*p*)	MAF	SNP effect	References
DOF	C2.27561819	A/G	2	27561819	3.5	0.29	1.99	^25, 42^
FLL	C2.27562491	A/T	2	27562491	3.7	0.14	2.89	^17, 42^
	C2.27562638	A/C	2	27562638	3.7	0.14	2.89	
	C2.27726456	G/A	2	27726456	3.1	0.27	2.11	
	C5.17545821	C/G	5	17545821	3.1	0.11	3.25	^17^
	C5.17545822	A/C	5	17545822	3.1	0.11	3.25	
	C5.20371666	C/A	5	20371666	3.1	0.11	3.25	
	C5.20537816	G/A	5	20537816	3.4	0.11	3.38	
FLW	C1.6814673	T/C	1	6814673	4.7	0.13	−0.5	—
	C1.6814709	A/G	1	6814709	4.7	0.13	−0.5	
	C1.6814725	A/G	1	6814725	4.7	0.13	−0.5	
	C1.6825210	T/C	1	6825210	5.1	0.11	−0.41	
	C1.6898417	G/T	1	6898417	5.5	0.15	0.13	
	C1.6904134	A/G	1	6904134	3.1	0.12	−0.35	
	C2.45151497	G/A	2	45151497	4.5	0.3	0.13	^17, 42^
	C3.30858639	G/A	3	30858639	4.6	0.06	−0.25	—
	C4.17271685	C/G	4	17271685	3.2	0.11	−0.2	—
	C4.17271782	C/T	4	17271782	3.2	0.11	−0.19	
	C4.17271815	A/G	4	17271815	3.2	0.11	−0.2	
	C6.2639715	G/C	6	2639715	3.2	0.11	0.13	—
	C7.26457626	G/A	7	26457626	3.4	0.24	−0.13	^25^
	C7.34109248	A/C	7	34109248	5.7	0.07	0.31	
	C8.29396204	C/T	8	29396204	5.4	0.06	−0.3	—
	C8.29469536	G/A	8	29469536	5.6	0.06	0.37	
	C9.37225457	C/T	9	37225457	17.3	0.12	0.16	—
	C9.37443288	C/A	9	37443288	16.5	0.11	−1.84	
	C9.37523364	C/A	9	37523364	6.0	0.12	−0.68	
	C9.38068016	C/T	9	38068016	16.5	0.11	−1.84	
	C9.47023159	A/T	9	47023159	4.6	0.09	0.19	
GY	C1.19584662	A/G	1	19584662	4.2	0.11	2.26	—
	C1.31690904	G/A	1	31690904	4.6	0.1	−3	
	C2.2290737	A/T	2	2290737	3.9	0.09	2.43	—
	C3.27957942	G/A	3	27957942	3.1	0.07	−2.48	^25^
	C3.28147097	G/A	3	28147097	3.1	0.07	−2.48	
	C3.29268216	T/A	3	29268216	3.1	0.07	−2.48	
	C3.32214003	G/C	3	32214003	3.1	0.07	−2.48	
	C3.32823330	C/T	3	32823330	3.1	0.07	−2.48	
	C3.35621706	C/T	3	35621706	3.4	0.08	−2.51	
	C3.50114070	A/G	3	50114070	6.8	0.07	4.05	
	C3.50516216	T/C	3	50516216	3.2	0.38	−1.34	
	C5.3047617	C/G	5	3047617	4.7	0.07	2.7	—
	C6.2476369	C/T	6	2476369	3.5	0.2	−1.47	—
	C7.12591441	G/A	7	12591441	4.3	0.11	2.89	—
	C7.14255739	C/T	7	14255739	3.4	0.15	−1.73	
	C7.19705515	A/C	7	19705515	6.1	0.13	6.18	
	C8.33295392	C/G	8	33295392	4.6	0.37	1.34	—
PanL	C2.29645716	G/A	2	29645716	3.4	0.22	1.57	^17, 58^
	C2.29645949	T/C	2	29645949	3.8	0.23	1.62	
	C3.14502439	C/T	3	14502439	3.0	0.39	−1.25	^25^
	C5.13976998	C/A	5	13976998	3.1	0.09	3.23	^17^
	C7.3618032	T/C	7	3618032	3.3	0.11	−2.07	^25^
	C8.4806303	T/C	8	4806303	3.3	0.11	−1.86	^25, 59^
	C9.54570214	G/T	9	54570214	3.2	0.12	1.83	^17, 25^
PedL	C9.14370304	A/C	9	14370304	4.1	0.13	2.11	—
	C9.54646405	C/A	9	54646405	3.5	0.19	1.74	
PH	C5.9294227	C/G	5	9294227	3.4	0.24	9.02	^17, 42^
	C5.17368448	G/C	5	17368448	3.0	0.12	11.32	
	C7.13010170	A/C	7	13010170	3.1	0.07	13.73	^17, 58^
TGW	C2.1884254	G/A	2	1884254	4.8	0.21	0.14	—
	C2.4114681	T/C	2	4114681	4.5	0.32	0.1	
	C3.4813917	T/C	3	4813917	3.7	0.23	0.1	^17, 25^
	C3.43310250	C/T	3	43310250	4.9	0.06	−0.25	
	C6.34654923	T/C	6	34654923	7.0	0.13	−0.21	^25^
	C8.23236893	C/A	8	23236893	3.0	0.07	0.13	—
	C8.34760860	A/C	8	34760860	4.2	0.36	0.1	
	C8.39522974	C/T	8	39522974	3.7	0.25	0.11	
	C9.37011889	A/G	9	37011889	8.3	0.08	−0.33	^59^
	C9.55718390	T/G	9	55718390	3.7	0.1	−0.19	
TM	C3.50460866	T/C	3	50460866	3.7	0.37	2.88	—
	C5.6559967	G/A	5	6559967	3.0	0.13	−3.5	—
	C6.34314915	G/A	6	34314915	3.5	0.41	2.58	—
	C7.18807581	A/G	7	18807581	3.2	0.06	−4.82	—
	C7.18869945	G/C	7	18869945	3.3	0.06	−4.88	
	C8.2541016	G/C	8	2541016	3.2	0.16	−3.33	—
	C9.10635590	T/C	9	10635590	3.2	0.2	3.02	—
TN	C4.8882993	G/A	4	8882993	3.1	0.22	0.49	^17, 25^
	C7.19587543	G/A	7	19587543	3.2	0.13	0.98	^25^
	C7.19696191	C/T	7	19696191	3.3	0.11	1.29	
	C7.19705515	A/C	7	19705515	3.6	0.13	1.37	
	C7.19718741	G/A	7	19718741	3.3	0.11	1.29	
	C9.37523364	C/A	9	37523364	3.1	0.12	0.91	^25^

### High confidence marker-trait association

To eliminate the false positive due to multiple testing, the MTAs were filtered following Bonferroni correction. Out of 81 MTAs, only seven MTAs could fulfill Bonferroni criteria, thus considered as high confidence MTAs (Table 3). These seven MTAs were associated with three traits including FLW (three), GY (two) and TGW (two). All the three MTAs associated with FLW were present on chromosome 9, *viz*., C9.37225457 (*p*-value 5.06 × 10^−18^), C9.37443288 (*p*-value 3.12 × 10^−17^) and C9.38068016 (*p*-value 3.12 × 10^−17^). Two MTAs associated with GY were present on chromosome 3 (C3.50114070, *p*-value 1.77 × 10^−7^) and chromosome 7 (C7.19705515, *p*-value 7.82 × 10^−7^). Similarly, two MTAs associated with TGW were found on chromosome 6 (C6.34654923, *p*-value 9.52 × 10^−8^) and chromosome 9 (C9.37011889, *p*-value 4.98 × 10^−9^). The above mentioned seven high confidence MTAs were further subjected to downstream analysis including the estimation of allele effect, identification of desirable allele and pyramiding effect of desirable alleles.

**Table 3:** List of Marker-trait associations (MTAs) fulfill the Bonferroni correction, with desirable allele and desirable allele effect. Desirable genotypes with desirable SNP alleles and phenotype may be used in foxtail breeding.

Trait	SNP	Chr.	Position	SNP alleles	Desirable allele	Desirable Allele effect	P-value	Desirable genotype
FLW	C9.37225457	9	37225457	C/T	T	0.9**	5.06E-18	F8, F34 (3.2 cm)
	C9.37443288	9	37443288	C/A	A	0.6**	3.12E-17	
	C9.38068016	9	38068016	C/T	T	0.6**	3.12E-17	
GY	C3.50114070	3	50114070	A/G	G	12.7**	1.77E-07	F14 (42.3 g)
	C7.19705515	7	19705515	A/C	C	11.5**	7.82E-07	
TGW	C9.37011889	9	37011889	A/G	A	0.1**	4.98E-09	D85, D76 (3.8 g)
	C6.34654923	6	34654923	T/C	T	0.1**	9.52E-08	

^**^0.01 level of significance in Kruskal–Wallis test.

### Allele effect and identification of desirable allele

The allele effect was determined for each allele of SNPs involved in seven MTAs involving three traits that fulfilled the Bonferroni criteria (Table 3). For all the three traits including FLW, GY and TGW, positive selection is required; thus, SNP alleles with positive allele effect were considered as desirable. For FLW, C9.37225457-T, C9.37443288-A and C9.38068016-T were found desirable; for GY, C3.50114070-G and C7.19705515-C were desirable; and for TGW, C6.34654923-T and C9.37011889-A were desirable to increase the trait values. The phenotype, with a desirable and undesirable allele of associated SNPs, were further tested using ‘Kruskal–Wallis test,’ which revealed significant difference for each phenotype (with and without desirable SNP allele) for all seven SNPs.

### Identification of putative candidate genes

Total 27 candidate genes were identified which were residing within 25 Kb regions (upstream and downstream) of seven high confidence MTAs for threes traits (FLW, GY and TGW), see Table 4, however, no associated SNPs were present within the gene. For three MTAs associated with FLW, total nine candidate genes were identified which found to encode AP2 domain, amino-oxononanoate synthase, ubiquitous protein, peptidases/proteases, pollen allergen1, arabinonate dehydratase/hydrolyase, etc. Similarly, ten candidate genes were identified in genomic regions harbouring two MTAs associated with GY; these genes encoded proteins involved in RBR family ring finger and IBR domain, translation initiation factor 3, pyridine nucleotide-disulfide oxidoreductase domain-containing protein 2, RNA recognition motif, LEA protein, glycosyltransferases, etc. For two MTAs associated with TGW, eight candidate genes were found involved in Sentrin/sumo-specific protease, DNA polymerase delta subunit 4, ATP-binding cassette transporter, etc. (Table 4).

**Table 4:** Putative candidate genes residing close vicinity (25 Kb either side) to associated SNPs.

Trait	Associated SNP	Transcript	Start	End	Strand	Description^a^
FLW	C9.37225457	Seita.9G323400.1	37216841	37217637	+	—
		Seita.9G323300.1	37206818	37208370	+	AP2 domain (AP2)
		Seita.9G323200.1	37197886	37201790	−	8-amino-7-oxononanoate synthase
	C9.37443288	Seita.9G323700.1	37427089	37428983	+	Protein of unknown function
		Seita.9G323800.1	37438152	37439633	−	Ancient ubiquitous protein
		Seita.9G323900.1	37444252	37445177	−	—
		Seita.9G324000.1	37445345	37446088	−	Ulp1 peptidase/protease
	C9.38068016	Seita.9G327100.1	38052985	38054536	+	Pollen allergen 1/DPBB_1
		Seita.9G327200.1	38083746	38088203	+	Arabinonate dehydratase/hydro-lyase
GY	C3.50114070	Seita.3G401100.1	50096532	50101244	−	RBR family ring finger and IBR domain-containing
		Seita.3G401200.1	50109418	50111773	+	—
		Seita.3G401300.1	50115178	50119847	+	Translation initiation factor IF-3
		Seita.3G401400.1	50120425	50125567	−	Pyridine nucleotide-disulfide oxidoreductase domain-containing protein 2
		Seita.3G401500.1	50128614	50130162	+	RNA recognition motif
		Seita.3G401600.1	50129252	50133460	−	ADP-ribosylation factor-like protein 2
	C7.19705515	Seita.7G094000.1	19688870	19690228	−	Late embryogenesis abundant protein 2
		Seita.7G094100.1	19693214	19697416	+	Glycosyltransferase
		Seita.7G094200.1	19701121	19703506	−	Aluminum-activated malate transporter 8
		Seita.7G094300.1	19728010	19729582	−	WDSAM1 protein
TGW	C9.37011889	Seita.9G321400.1	36991641	36994547	+	Sentrin/sumo-specific protease
		Seita.9G321500.1	36997661	37000034	−	HD-ZIP protein N terminus
		Seita.9G321600.1	37005554	37011470	−	U-box domain-containing protein 35-related
		Seita.9G321700.1	37016333	37018688	−	Ulp1 peptidase/Ulp1 protease
	C6.34654923	Seita.6G234400.1	34633343	34633845	−	DNA polymerase delta subunit 4
		Seita.6G234500.1	34641134	34645923	+	—
		Seita.6G234600.1	34655584	34665499	+	ATP-binding cassette transporter
		Seita.6G234700.1	34677410	34679017	+	—

^a^‘—’represents no annotation available.

### Pyramiding effect and desirable genotypes

**Figure 4:**
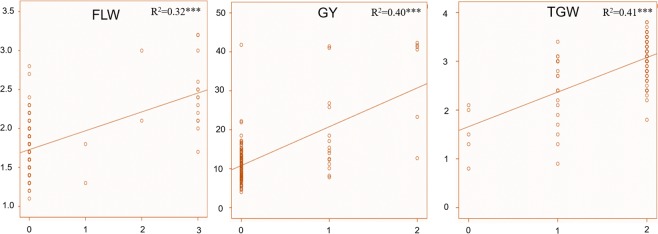
Linear regression analysis for phenotype (dependent variable) and number of desirable SNP alleles (independent variable). R^2^ = regression coefficient; ***represents 0.0001 level of significance. x-axis represents number of desirable SNP alleles and y-axis represents phenotypic values.

The pyramiding effect of desirable alleles (including more than one SNP associated with the trait) was calculated for above mentioned three traits involving seven MTAs (that fulfilled Bonferroni criteria; Fig. 4). Analysis of pyramiding effect showed that an increase in a number of desirable alleles significantly affects the trait value. For instance, two SNPs associated with GY; genotypes with no desirable allele had a mean GY = 11.0 g, while the genotypes with one desirable allele had a mean GY = 18.4 g and genotypes with two desirable alleles had a mean GY = 33.6 g. The difference between mean GY with two, one and zero desirable alleles was highly significant (r^2^ = 0.40; *p* ≤ 0.000). A similar trend was observed for TGW. Mean TGW values of genotypes with two (3.1 g), one (2.4 g) and without any desirable alleles (1.5 g) showed significant difference among them (r^2^ = 0.41; *p* ≤ 0.000). For FLW, genotypes with three desirable alleles had significantly wider flag leaf (2.4 cm) than genotypes with no desirable allele (1.7 cm) and one desirable allele (1.5 cm). However, no significant difference was observed for FLW in genotypes with two desirable alleles and three desirable alleles.

Further, most desirable genotypes with a maximum number of the desirable allele for each of the above mentioned three traits (FLW, GY and TGW) were identified (Table 3). For FLW, two genotypes including F8 and F34 were identified to have desirable alleles of three associated SNPs, and also showed higher traits value (3.2 cm). For GY, one genotype (F14; 42.3 g) was identified to have a desirable allele of two associated SNPs. Similarly, for TGW, two genotypes (D85, D76) were identified to have higher TGW and desirable alleles for two SNPs.

## Discussion

GWAS has always been a potential approach for genetic dissection of complex traits, and it has been successfully utilized in a number of crops including wheat, rice, pearl millet, maize and cotton^22,28–32^. However, in foxtail millet, only a couple of studies are available where GWAS was utilized to identify genomic regions controlling traits of interest^25,26^. Genetic diversity of panel is important prerequisites for GWAS and has been conducted in our earlier study^26^ which suggested that the panel is diverse. Out of 142, 89 genotypes were collected from different parts of India including Andhra Pradesh, Bihar, Tamilnadu, Jammu & Kashmir, Karnataka, Maharashtra, Madhya Pradesh, Rajasthan, Uttara Khand and West Bengal; remaining 53 genotypes were exotic and belonged to nine different countries (for details see Gupta *et al*.^26^). Further, population structure creates confounding in GWAS results. Thus it is important to conduct population structure analysis. Our earlier study^26^ suggested that there were five subpopulations in the panel.

Further, size and trait diversity of population also affects the power of GWAS^33^. In the present study, utilization of 142 genotypes which are sufficiently diverse suggested their suitability for GWAS. The size of the population used in the present study is slightly small but found to be comparable with GWAS conducted in other cereal crops including millets^34–36^. It has been suggested that small population size may be inefficient for the identification of significant associations with minor effect^37^. In the present study, we have identified significant MTAs even after implementing the most stringent Bonferroni correction. This suggested that the population size is good enough for GWAS; although, we agree that a larger population may lead to the identification of more MTAs. Descriptive statistics and frequency distribution (Supplementary Table 1; Supplementary Fig. 1) suggested that the panel had enough variability for each of the ten traits. Another important prerequisite of GWAS is the high-density marker^19,20,38,39^. In the present study, GBS enabled the development of a high-density genotyping data with ~10 K SNPs to conduct GWAS for agronomic traits.

GWAS is based on LD which itself is affected by several factors including physical linkage, recombination, selection, genetic drift, etc.^40^. Furthermore, self-pollinating crops show stronger LD than cross-pollinating crop^40–42^. Higher recombination rate causes faster LD decay which ultimately results in higher mapping resolution. It is well understood that the recombination rate varies through genome^43,44^, and thus, some genomic regions have been identified as recombination hot-spots, where recombination rate is higher and vice-versa^45^. In foxtail millet, genome-wide LD is reported as 100 Kb^25^. In the present study, we estimated the genome-wide LD decay as well as for each of the nine chromosomes individually. It was observed that the decay distance varied across different chromosomes (76 Kb on chromosome 6 to 357 Kb on chromosome 4); genome-wide decay distance was found to be 177 Kb, which is well at par with the earlier study^25^. The rapid LD decay suggested that the population used in the present study is sufficiently diversified and suitable to conduct GWAS. Thus, the rapid LD decay and higher mapping resolution also made the study useful for cloning of QTLs.

A major limitation of GWAS is false positives that arise due to population structure. However, false positives may be reduced with the use of statistical models^46,47^. Although correction for population structure plays a vital role in reducing false positives, overcorrection may lead to false negative results^22^. Therefore, we initially tested the model fitting for population structure correction. Q-Q plots of all the ten traits showed the proper distribution of observed *p-*values over expected, which suggested that the association model used in this study is the best fit and maximized the confidence of GWAS results. Further, false positives may also arise due to multiple testing, because in each test using single SNP there is at least 5% error and with an increase in the number of SNPs (i.e., the number of tests) overall experimental error increases. Several statistical tools are available for multiple testing correction^27^. In the present study, we also applied corrections for multiple testing (e.g., Bonferroni correction) during GWAS. Here, we observed that out of 81 MTAs (associated with ten traits) only seven MTAs (associated with three traits) qualified Bonferroni correction. Although multiple testing corrections are important to reduce false positive, it becomes highly stringent due to the use of thousands of makers; and may lead to false negatives. Thus, FDR is also considered as a tradeoff and escaped detection of genuine MTAs in earlier studies^22,48,49^. In our study, we also observed that even MTAs with a very significant *p*-value (10^−5^) could not qualify multiple testing correction criteria. Thus, all the 81 MTAs may not be false, and thus, need further validation; however, seven MTAs those fulfilled the multiple testing correction criteria had more confidence.

It is widely known that most of the traits are complex and are controlled by a large number of genes/QTLs^15,17,19,20,50,51^. In foxtail millet, linkage-based QTL mapping has been conducted for a number of agronomic traits such as days to heading, peduncle length, grain weight biomass, spikelet, yield, etc.^15–17,52^ using biparental mapping population. The identification of 81 MTAs in the present study adds to the existing knowledge of the genetic architecture of traits considered. Further, single locus analysis furnishes biased results since the background is not considered in this approach. Given this, we have conducted multi-locus analysis by considering the background genome as cofactor using recently developed FarmCPU approach^53^. Interestingly, out of 81 MTAs, 60 were present on the same chromosome where QTL/s for the same traits were reported in earlier studies^17,25,42^ (Table 2).

Seven high confidence MTAs were identified for three important agronomic traits including FLW, GY and TGW may prove useful in foxtail millet breeding program through marker-assisted selection after validation. For validation, linkage based interval mapping or joint linkage-LD mapping may be conducted using biparental mapping population or specialized populations (NAM, MAGIC). The above mentioned seven high confidence SNPs also found crucial in identifying important candidate genes underlying these traits. Candidate genes present close vicinity of associated SNPs identified in the present study may be validated in the future so that can be deployed in breeding. For validation, one can use candidate gene-based association mapping using large population, or functional characterization through RNAi, VIGS, etc. Identification of desirable alleles of these MTAs will enable their efficient utilization in crop improvement programs. Interestingly, the significant pyramiding effect of multiple MTAs for the single trait in our study suggested that the associated SNPs may combine to improve the trait substantially. For three traits (FLW, GY and TGW), three desirable genotypes were identified with a maximum number of desirable alleles. These genotypes may be used as a donor in foxtail millet breeding program. Intercrossing of these genotypes may combine desirable traits to develop improved high yielding foxtail genotype.

In foxtail millet, studies are available where GWAS has been conducted for agronomic traits using few SSRs^26^ and million SNPs^25^. The present study provides better resolution of trait mapping using 10 K SNPs as compared to an earlier study^26^. Jia *et al*.^25^ conducted GWAS for 47 agronomic traits using 916 accessions and 0.8 million SNPs developed through whole-genome sequencing. The present study, where GWAS was conducted for ten agronomic traits (nine were common with Jia *et al*.^25^, TM was not studied in Jia *et al*.^25^) with lesser SNPs and accessions, may be questioned for its novelty. There are two parameters which made present study novel- (i) utilization of different accessions with different genetic background and (ii) phenotyping in different environments. These two parameters enabled us to identify some novel genomic regions associated with agronomic traits (Table 2). For example, two high confidence SNPs, one each associated with GY (C6.2476369) and TGW (C9.37011889) were identified on chromosome 6 and chromosome 9, respectively, in the present study. However, Jia *et al*.^24^ did not identify any SNPs associated with GY and TGW on chromosomes 6 and 9, respectively.

The present study explored the genetic architecture of ten agronomic traits using LD based GWAS exploiting historical recombination in a natural population. The population used in the present study showed a wide range of variability for traits studied. Further, ddRAD-seq provided high-density genotypic data which is a pre-requisite for GWAS. Our study led to the identification of 81 MTAs for agronomic traits including some novel MTAs and provided better insights into the genetic architecture of traits. Significant pyramiding effect of associated SNPs with the same trait suggested their potential utilization in foxtail breeding. The desirable alleles and genotypes identified in this study will be useful in crop improvement programmes.

## Materials and Methods

### Plant material and phenotyping

The phenotypic data of 142 foxtail millet genotypes previously reported by Gupta *et al*.^26^ was used in the present study. Precisely, the genotypes were phenotyped for ten yield contributing agronomic traits for three consecutive years (2009–2011) at the research fields of National Institute of Plant Genome Research (NIPGR), New Delhi, India, in a randomized complete block design with three replications. Mean data over the years of each of the 10 traits were utilized during the present study. The traits included days to flowering (DOF), plant height (PH), tiller number (TN), flag leaf length (FLL), flag leaf width (FLW), peduncle length (PedL), panicle length (PanL), tiller maturity (TM), grain yield (GY), and 1000 grain weight (TGW). Descriptive statistics, frequency distribution and Pearson’s correlation coefficient between all possible trait-pairs were analyzed using SPSS v17.0 software.

### ddRAD sequencing and SNP calling

DNA was extracted from one-month-old leaf samples of 142 genotypes using the CTAB method^54^. The DNA samples were RNase treated to remove RNA contamination, and the quality and quantity were checked on 1% agarose gel and NanoDrop 1000 (Thermo Scientific). For genotyping, Double Digest Restriction Associated DNA (ddRAD) sequencing approach was used^55^, and sequencing was done using Illumina Hiseq4000 (AgriGenome Labs Pvt Ltd, Hyderabad, India). Raw Fastq reads were demultiplexed allowing one mismatch to obtain reads for each sample. Data were filtered on the basis of RAD TAGs. Filtered reads were then subjected to 5′ and 3′ base trimming. Illumina 5′ and 3′ adapter sequences were also removed. Paired-end alignment was performed using Bowtie2 (version 2-2.2.9) program with default parameters to the reference genome (http://genome.jgi.doe.gov). The aligned samples and the reference genome sequence are used for variant calling using default settings of SAMtools version 0.1.18.

### Linkage disequilibrium

LD (in terms of r^2^) analysis was performed for the whole genome as well as individually for each of the nine chromosomes using window size 50 with the help of software TASSEL v5.0. To estimate LD decay, non-linear regression curve was utilized^56^, and LD decay distance was estimated as the physical distance between SNPs where average r^2^ reduced to half of the maximum LD value.

### Marker-trait associations

SNPs with <30% missing data and >5% minor allele frequency were utilized for GWAS. All the 142 genotypes used for GWAS were having <30% missing genotypic data. For the association test, a recently developed method called Fixed and random model Circulating Probability Unification (FarmCPU^53^) was used. This method is highly efficient and also eliminates confounding issues arising due to population structure, kinship, multiple testing correction, etc. This method utilizes both Fixed Effect Model (FEM) and a Random Effect Model (REM), iteratively. REM estimated pseudo-quantitative trait nucleotides (QTNs) and FEM tested marker using pseudo QTNs as covariates. First, three components identified through principal component analysis (PCA) using TASSEL v5.0 were included as a covariate in the association test model. SNP with *p*-value < 0.001 declared as significant MTAs. Bonferroni-corrected *p*-value threshold was set as 0.01. To show the model fitting (accounting for population structure), quantile-quantile (Q-Q) plots were also analyzed. The Q-Q plot showed the distribution of observed and expected *p*-values (association test statistics). Desirably in case of appropriate model fitting, Q-Q plots should show a solid line (i.e., the distribution of observed *p*-value is similar to expected one) represented no biasness; and sharp curves at the end which represented a small number of true associations among thousands of unassociated SNPs. The extent of deviation of curve end from the diagonal is the measure of the power of test statistics.

### Allele effect and pyramiding effect of desirable alleles; identification of desirable genotypes

Phenotypic effect (a_i_) of each allele of SNPs (significantly associated with trait following Bonferroni correction) was estimated following Zhang *et al*.^57^. Kruskal–Wallis test was performed to identify whether the alleles differ considerably for the associated traits. Subsequently, favourable alleles were identified for each of the trait considered according to the breeding objective. For traits with negative selection, a_i_ < 0 was considered as desirable allele; while, for a trait with positive selection, a_i_ > 0 was considered as a desirable allele.

The pyramiding effect was estimated in the case where more than two SNPs were found to be associated with the same trait (after Bonferroni correction). To determine the pyramiding effect, linear regression was performed using the number of desirable SNP alleles for traits (independent variable) and corresponding trait values of the genotypes that contained different numbers of desirable SNP alleles (dependent variable). Genotypes with a maximum number of desirable alleles and desirable phenotype were considered as a desirable genotype.

### Identification of putative candidate genes

To identify putative candidate genes residing at the close vicinity of high confidence SNPs, the associated SNPs were mapped to the reference genome *Setaria italica* v2.2 (https://phytozome.jgi.doe.gov/pz/portal.html#!info?alias=Org_Sitalica). Transcripts present within 25 Kb regions from both sides of associated SNPs were fetched along with their description.

## Supplementary information

Supplementary information

## Data Availability

The datasets supporting the conclusions of this article are included within the article and its Additional files.
