# Supplementary material for: Genome-Wide Association Study of Major Agronomic Traits in Foxtail Millet (Setaria italica L.) Using ddRAD Sequencing
Source: Sci Rep. 2019 Mar 22;9:5020. doi: 10.1038/s41598-019-41602-6 (PMC6430830; doi:10.1038/s41598-019-41602-6)
Supplement: Supplementary file 1 — Supplementary information [file 41598_2019_41602_MOESM1_ESM.pdf]

Genome-Wide Association Study of Major Agronomic Traits in Foxtail Millet (*Setaria italica* L.) Using ddRAD Sequencing

Vandana Jaiswal[1], Sarika Gupta[1], Vijay Gahlaut[2], Mehanathan Muthamilarasan[3,4], Tirthankar Bandyopadhyay[3], Nirala Ramchiary[1], Manoj Prasad[3]*

[1]School of Life Science, Jawaharlal Nehru University, New Delhi 110067, India

[2]Department of Plant Molecular Biology, University of Delhi South Campus, New Delhi110021, India

[3]National Institute of Plant Genome Research, New Delhi 110067, India

[4]ICAR-National Research Centre on Plant Biotechnology, LBS Centre, Pusa Campus, New Delhi 110012, India

*__Corresponding author__

Manoj Prasad

National Institute of Plant Genome Research

Aruna Asaf Ali Marg

New Delhi 110067, India

Tel: 91-11-26741612, Fax: 91-11-26741658

E-mail: manoj_prasad@nipgr.ac.in

Supplementary Figure 1. Frequency distribution of 10 agronomic traits in 142 foxtail millet genotypes used for GWAS. The x-axis represents the trait value and the y-axis represents frequency. Days to flowering (DOF), plant height (PH), tiller number (TN), flag leaf length (FLL), flag leaf width (FLW), peduncle length (PedL), panicle length (PanL), tiller maturity (TM), grain yield (GY), and 1000 grain weight (TGW).

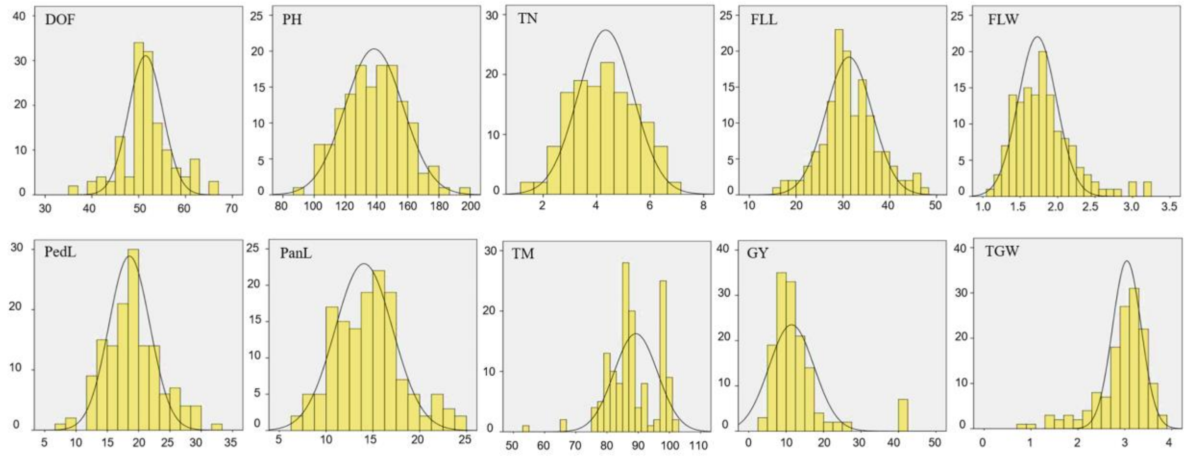

Supplementary Figure 2. Linkage disequilibrium (LD) decay plots for nine foxtail millet. x-axis represents distance (bases) between SNPs and y-axis represents LD value (r^2; 0.0, 0.2, 0.4, 0.6, 0.8, 1.0). Horizontal and vertical lines represent half LD and LD decay distance respectively.

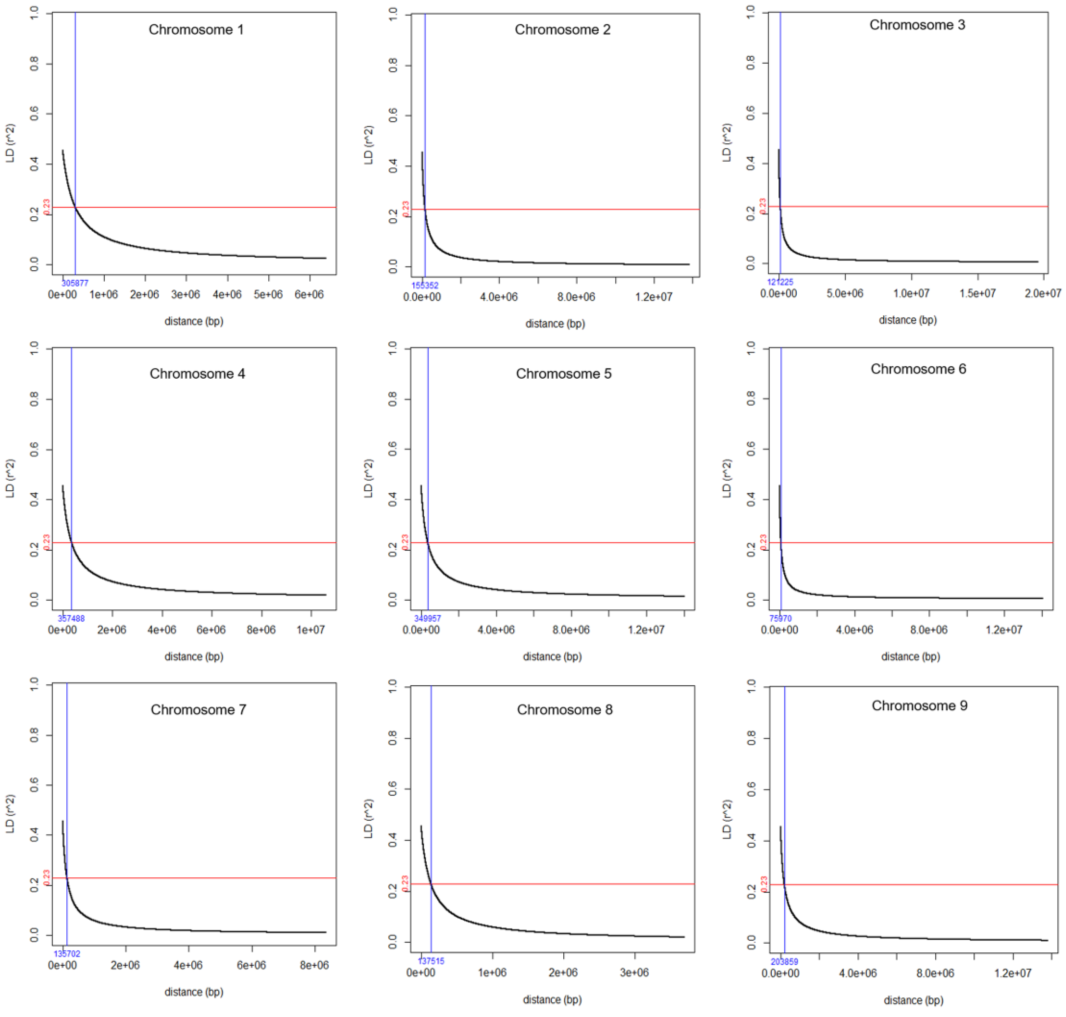

Supplementary Table 1. Descriptive statistics including minimum, maximum, mean and standard deviation (SD) for ten agronomic traits in 142 foxtail millet genotypes.

Trait	Minimum	Maximum	Mean	SD
DOF (days)	36.0	65.0	51.4	5.6
PH (cm)	89.7	194.0	138.4	19.6
TN (number)	1.4	6.8	4.3	1.2
FLL (cm)	16.6	47.3	31.4	6.0
FLW (cm)	1.1	3.2	1.8	0.4
PedL (cm)	6.9	31.7	19.0	4.4
PanL (cm)	6.6	24.4	14.6	3.7
TM (days)	54.0	101.0	87.8	8.0
GY (g)	4.0	42.3	12.8	7.8
TGW (g)	0.8	3.8	2.9	0.6

Supplementary Table 2. Karl Pearson's coefficient of correlation for all possible pairs involving ten traits.

Traits	DOF	PH	TN	FLL	FLW	PedL	PanL	TM	GY	TGW
**DOF**	1	.124	.059	-.056	.025	-.085	-.150	.200*	.054	.097
**PH**		1	.090	.275**	.082	.188*	.258**	.380**	.043	.065
**TN**			1	.113	.103	-.207*	.125	.108	.381**	-.220**
**FLL**				1	.471**	-.028	.436**	-.009	.331**	-.214*
**FLW**					1	-.246**	.361**	-.113	.537**	-.433**
**PedL**						1	.136	.146	-.267**	.323**
**PanL**							1	-.055	.436**	-.238**
**TM**								1	-.132	.276**
**GY**									1	-.458**
**TGW**										1

* and ** represent 0.05 and 0.001 level of significance.
